# Physicochemical and Mechanical Properties of Non-Isocyanate Polyhydroxyurethanes (NIPHUs) from Epoxidized Soybean Oil: Candidates for Wound Dressing Applications

**DOI:** 10.3390/polym16111514

**Published:** 2024-05-27

**Authors:** Maria Morales-González, Manuel F. Valero, Luis E. Díaz

**Affiliations:** 1 Doctoral Program in Engineering, Faculty of Engineering, Universidad de La Sabana, Chía 140013, Colombia; mariamorgon@unisabana.edu.co; 2 Energy, Materials and Environmental Group (GEMA), Faculty of Engineering, Universidad de La Sabana, Chía 140013, Colombia; manuelvv@unisabana.edu.co; 3 Bioprospecting Research Group (GIBP), Faculty of Engineering, Universidad de La Sabana, Chía 140013, Colombia

**Keywords:** non-isocyanate polyhydroxyurethanes, soybean oil, wound dressing

## Abstract

Only 0.1% of polyurethanes available on the market are from renewable sources. With increasing concern about climate change, the substitution of monomers derived from petrochemical sources and the application of eco-friendly synthesis processes is crucial for the development of biomaterials. Therefore, polyhydroxyurethanes have been utilized, as their synthesis route allows for the carbonation of vegetable oils with carbon dioxide and the substitution of isocyanates known for their high toxicity, carcinogenicity, and petrochemical origin. In this study, polyhydroxyurethanes were obtained from carbonated soybean oil in combination with two diamines, one that is aliphatic (1,4-butadiamine (putrescine)) and another that is cycloaliphatic (1,3-cyclohexanobis(methylamine)). Four polyhydroxyurethanes were obtained, showing stability in hydrolytic and oxidative media, thermal stability above 200 °C, tensile strength between 0.9 and 1.1 MPa, an elongation at break between 81 and 222%, a water absorption rate up 102%, and contact angles between 63.70 and 101.39. New formulations of bio-based NIPHUs can be developed with the inclusion of a cycloaliphatic diamine (CHM) for the improvement of mechanical properties, which represents a more sustainable process for obtaining NIPHUs with the physicochemical, mechanical, and thermal properties required for the preparation of wound dressings.

## 1. Introduction

There is growing demand for biomaterials, especially polyurethanes, for tissue engineering that can mimic tissue properties and behavior. Even though different polyurethanes have been proposed and investigated, with many being commercially available, less than 0.1% of the total polyurethanes currently available on the market come from renewable sources [1]. Common synthesis routes for polyurethanes involve the use of polyols and isocyanates [2]. The source of these monomers is petrochemical [3], and isocyanates have been found to be toxic, especially aromatic isocyanates, due to their degradation products [4].

One of the areas in which polyurethanes have been used is wound dressing applications, as they have advantages such as biocompatibility, mechanical properties similar to those of human skin, and flexibility [5,6]. However, the currently available wound dressings present disadvantages in the treatment of chronic wounds, including poor antibacterial activity, poor anti-inflammation activity, differences in mechanical strength from human skin, limited ability to absorb exudates, and inadequate gas exchange [5,7], which affect the healing process.

Film dressings has a better relationship with the adjacent tissue, causing less damage upon removal [8]. In addition, they achieve higher antibacterial activity compared to hydrogels and hydrocolloids [8,9]. Although some dressings generated with different techniques, such as electrospun nanofibers, have great advantages such as compatibility or antibacterial activity, in the case of nanofibers, by promoting cell adhesion, they result in greater damage to the adjacent tissue [10]. Also, film dressings present higher chemical resistance required for biomedical applications [11].

Although, currently, there is a wide range of products for this purpose, so far, an ideal dressing has not been created, so the search for a material that will accelerate the healing process and help to reduce the development of chronic or non-healing wounds, which represent a high burden on the health system, continues.

For this reason, recent research has focused on new polyurethane synthesis routes that allow for the incorporation and use of bio-based monomers and the substitution of isocyanates, and polyhydroxyurethanes (PHUs) are promising materials [1]. One of the synthesis routes comprises the use of carbon dioxide (CO_2_) and the incorporation of bio-based monomers from renewable sources, such as vegetable oils [12]. In this route, cyclic carbonates are ring-opened with amines, whereby an alkoxyl ion is produced, which forms a hydroxyurethane bond upon reaction with amines, leading to the production of PHUs [13]. For the generation of cyclic carbonates, vegetable oils can be used, as they present several advantages due to their chemical composition; vegetable oils have a double bond that allow for different chemical modifications, which generate a wide variety of polymers with diverse mechanical, physicochemical, and biological properties, thus allowing their use in various applications [14]. Another advantage of this synthesis route is the 100% atomic reaction, wherein CO_2_ acts as an aprotic and reactive solvent [12].

Soybean oil has been used in the synthesis of polyurethanes due to the presence of unsaturated fatty acids in its chain, which are susceptible to chemical modifications, such as epoxidation and carbonation. Its application allows the characteristic urethane bond to form. Although the synthesis route using soybean oil generates five-carbon cyclic carbonates, which are less reactive than those with six carbons, the renewable characteristic of soybean oil allows for the incorporation of more sustainable production processes. Additionally, soybean oil is a low-cost raw material and has been proven to be biocompatible in biomedical applications [15].

Specifically for wound dressings, Gholami and Yeganeh generated PHUs from soybean oil crosslinked with epichlorohydrin, with the inclusion of cationic azethidinium groups to improve the antibacterial properties of these dressings. The mechanical properties were a tensile strength of 6 MPa, an elongation at break of 200%, and water absorption of 30%, and these dressing were non-cytotoxic to L929 fibroblasts, generating a moist environment suitable for low to moderately exuding wounds [16].

Therefore, the search for biomaterials from renewable sources that can be used in more sustainable synthesis routes is crucial. The aim of this investigation was to determine the physicochemical, mechanical, and thermal properties of bio-based NIPHUs obtained from modified soybean oil with the incorporation of a cycloaliphatic diamine in the chain to increase the mechanical properties; these bio-based NIPHUs were generated to be potential candidates for wound dressing applications.

## 2. Materials and Methods

### 2.1. Materials

Epoxidized soybean oil (ESBO) was purchased from MPQ MapriQuim SAS (Bogotá, Colombia). Tetrabutylammonium bromide (TBABr), 1,4 butadiamine (BDA), and 1,3-cyclohexanobis(methylamine) (CHM) were procured from Sigma-Aldrich Co., LLC (Saint Louis, MO, USA). Additionally, 30% hydrogen peroxide and sodium bicarbonate were purchased from PanReac AppliChem ITW Reagents (Darmstadt, Germany). CO_2_ was purchased from Messer (Bogotá, Colombia), and phosphate buffer from VWR (Radnor, PA, USA).

### 2.2. Soybean Oil Carbonatation

To obtain carbonated soybean (CSBO), the procedure developed by Guzmán et al. (2017) was followed. ESBO and the catalyst TBABr at 5% mol with respect to the epoxide content were placed in a 160 mL high-pressure reactor (Parr Instrument Company, Moline, IL, USA) at 105 °C. When the desired temperature was reached, CO_2_ was continuously injected into the mixture at a pressure of 6.0 MPa. The reaction was run for 24 h at 500 rpm [17]. When the reaction was completed, the mixture was cooled to room temperature (25 °C).

### 2.3. Synthesis of Non-Isocyanate Polyhydroxyurethanes (NIPHUs)

NIPHUs were obtained based on the methodology proposed by Raden Amirah et al., 2013. Briefly, the obtained CSBO was heated to 60 °C. Then, the amines BDA and CHM were added in different proportions (Table 1) to reach a 1:2 molar ratio of cyclocarbonate to amine. The mixture was left under agitation at 60° C for 5 min, and then transferred to a mold and left to cure at 90 °C for 12 h [18].

### 2.4. Characterization

The characteristic chemical structures of ESBO, CSBO, and NIPHUs were evaluated via Fourier-transform infrared (FTIR) spectroscopy using an ATR-FTIR spectrometer (Agilent Cary 630 FTIR Spectrometer, Santa Clara, CA, USA) in the range between 400 and 4000 cm^−1^. The spectra were obtained at a spectral resolution of 4 cm^−1^ for an average of 24 scans. The molecular structures of products of carbonation reactions were analyzed via proton nuclear magnetic resonance to obtain ^1^HNMR and ^13^CNMR spectra using a Bruker Avance Neo 400 MHz spectrometer, Billerica, Massachusetts, USA and CDCl3 as the solvent.

### 2.5. Swelling

Sample disks (6 mm in diameter) were immersed in 1.5 mL of distilled water and incubated for 21 days at 37 °C [19]. The water absorption was calculated after 1, 2, 3, 5, 6, 24, 26, 28, 30, 48, 50, 52, 144, 168, 216, 288, 360, 456, and 504 h, with the initial weight (Wo) and final weight (Ws), according to the following equation:

(1) $$Water\;absoption\;rate\left(\%\right)=\frac{\left(w_{t}-w_{i}\right)}{w_{i}}\times 100\%$$

### 2.6. Contact Angle

The hydrophilic characteristic of the obtained NIPHUs was measured via the sessile drop method using an OCA20 device (Data Physics, Filderstadt, Germany) with deionized water at 25 °C. At least 15 measurements were taken per sample [20].

### 2.7. Hydrolytic and Oxidative Degradation

Degradation was evaluated in terms of the mass loss. Hydrolytic degradation was performed in phosphate buffer (PBS 1×: 137 mM NaCl, 10 mM phosphate, and 2.7 mM KCl) at a pH of 7.4. The oxidative degradation was evaluated in a 3% H_2_O_2_ solution [21]. The NIPHU samples (between 20 and 60 mg) were immersed in 2 mL of each solution for 21 days at 37 °C. Their weight was recorded every three days.

### 2.8. Mechanical Test

Stress–strain tests were performed using a DMA 850 (TA Instruments, New Castle, DE, USA), wherein the maximum stress, percentage of elongation, and Young’s modulus of the PHUs were determined (following the ASTM D638-10 [22]). A 5 kN-load cell with a jaw displacement speed of 25 mm min^−1^ was used [19]. Three specimens of 20 mm × 5 mm × 0.25 mm (length × width × thickness) were tested.

### 2.9. Thermal Characterization

The thermal stability of the obtained NIPHUs was evaluated with thermogravimetric analysis (TGA) in the range of 25 °C to 600 °C under a nitrogen atmosphere at a heating rate of 25 °C min^−1^.

### 2.10. Statistical Analysis

All experiments were performed at least three independent times. The data were reported as the mean ± standard deviation and were analyzed using one-way analysis of variance (ANOVA) to determine the statistical significance. A *p*-value of less than 0.05 was considered significant. Tukey’s multiple comparison test was performed to determine significant differences between pairs.

## 3. Results

**Figure 1:**
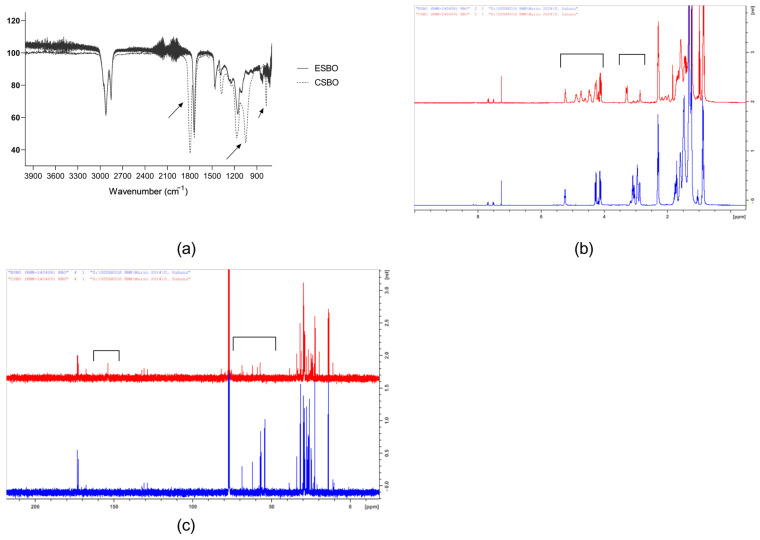
(**a**) FTIR spectra of ESBO and CSBO; (**b**) ^1^H RMN spectra of ESBO (blue) and CSBO (red); (**c**) ^13^C RMN spectra of ESBO (blue) and CSBO (red).

Epoxidized soybean oil reacted with CO_2_ and TBABr to generate carbonated soybean oil. From the FTIR spectra of the ESBO and CSBO, it is evident that a conversion of the epoxy groups, with a peak at 834 cm^−1^, to cyclic carbonate groups occurred, with the appearance of new signals at 1046, 1200, and 1801 cm^−1^ (Figure 1a). Similarly, the ^1^H RMN spectrum presented in Figure 1b indicates that the signals of the epoxy groups in the ESBO at 2.80–3.20 ppm disappeared in the CSBO, with the appearance of new signals at 4.45–5.10 ppm, corresponding to the cyclic carbonate groups [23,24]. Additionally, the ^13^C RMN spectrum presents a signal at 155 ppm due to the carbonyl group of cyclic carbonates in CSBO and indicates the disappearance of the signals at 54.16 and 56.75 ppm, corresponding to the epoxy groups [23].

The following reactions occurs for the carbonatation and synthesis of the NIPHUs.

$$\mathrm{ESBO}+{\mathrm{CO}}_{2}\;\begin{array}{c} \mathrm{TBABR}\\ \to \\ 105\;{}^{\circ}C \end{array}\;\mathrm{CSBO}\;\mathrm{CSBO}+\mathrm{BDA}+\mathrm{CHM}\;\overset{60\;{}^{\circ}C}{\to }\;\mathrm{NIPHU}$$

**Figure 2:**
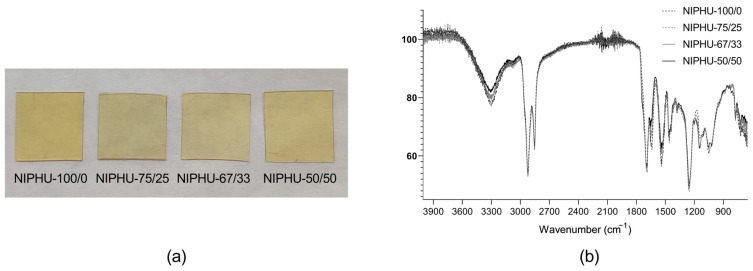
(**a**) NIPHU films synthesized from CSBO and different combinations of BDA and CHM; (**b**) FTIR spectra of synthetized NIPHU films.

Afterwards, NIPHUs were synthetized from CSBO, 1,4 butadiamine (BDA), and 1,3-cyclohexanobis(methylamine) (CHM). However, the synthesis of NIPHUs with a content of CHM over 50% resulted in brittle materials that broke during the demolding process; therefore, these NIPHUs were not characterized (Figure 2a). The disappearance of the 1801 cm^−1^ peak during the reaction and the appearance of the peaks at 1689, 1536, and 3309 cm^−1^ corresponding to the stretching of urethane carbonyl, the combination of C-N stretching/N-H bending, and the urethane N-H/hydroxyl OH stretching vibrations, respectively (Figure 2b), confirm that NIPHUs were obtained. Additionally, there was C–N bond stretching at around 1250 cm^−1^ and stretching vibrations of the C–O bond at 1141 cm^−1^ [25,26]. No differences were observed in the FTIR spectra of the synthetized NIPHUs.

The hydrophilicity or hydrophobicity of the obtained NIPHUs were evaluated by measuring the contact angle degree and water absorption rate (Figure 2). The surface wettability of these NIPHUs changed with the inclusion of CHM in the chains. With a lower content of CHM, a more hydrophilic behavior with a contact angle of 63.70 (NIPHU-50/50) was observed compared to the contact angle of 101.39 of the NIPHU without CHM (NIPHU-100/0). However, the water absorption rate was similar for NIPHUs with or without CHM, with a maximum absorption over 456 h (19 days) after equilibrium was reached at 102.88% and 104.29%, respectively (Figure 2b). No mass loss was observed within the 21 days of analysis for all the NIPHUs synthetized from CSBO and BDA/CHM in the hydrolytic and oxidative medium.

**Figure 3:**
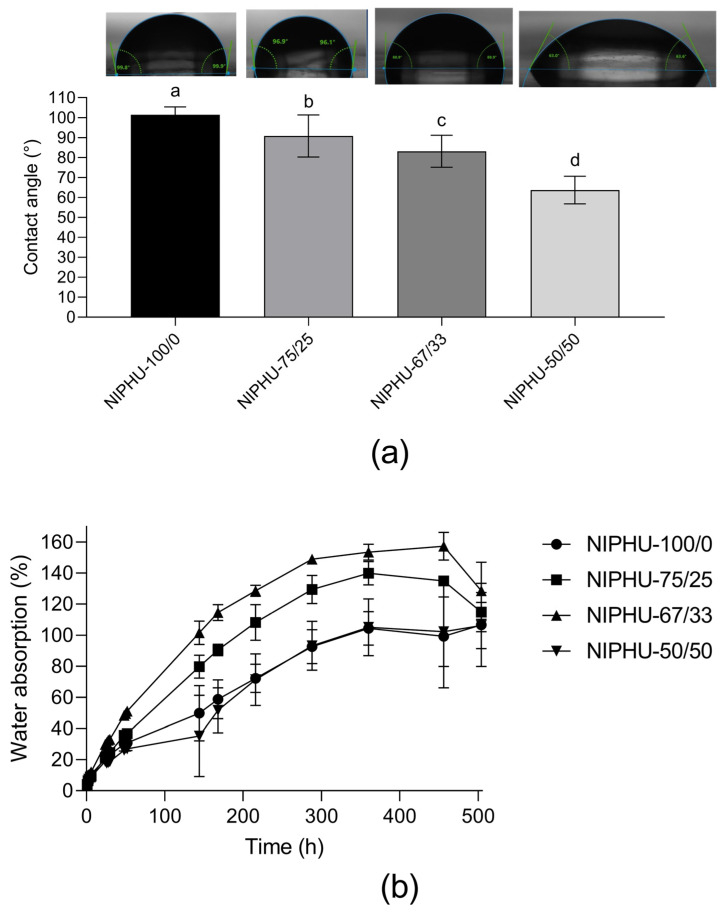
(**a**) Contact angle measurements, mean ± SD (*n* = 15), According to the analysis of variance and Tukey’s pairwise comparison test, means that do not share the same letter are significantly different (*p* < 0.05).; (**b**) water absorption rate (%) over 192 h, mean ± SD (*n* = 3).

**Figure 4:**
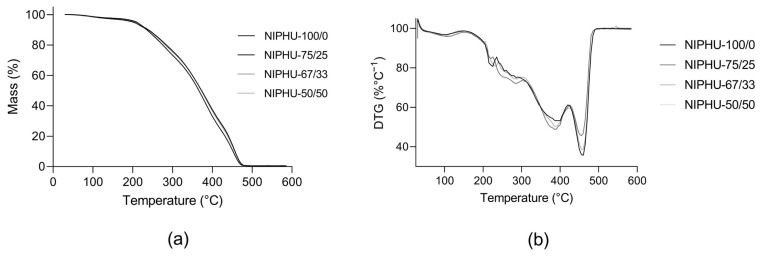
Thermograms of the synthesized NIPHUs: (**a**) thermogravimetric (TG) curve and (**b**) derivative of the thermogravimetric (DTG) curve.

The thermograms of the NIPHUs in Figure 3 display a three-stage weight loss process (Figure 4). The NIPHUs showed stable behavior up to 200 °C, and their degradation occurred after 350 °C [27].

The developed NIPHUs exhibited a tensile strength in the range of 0.5 to 1.13 MPa (Table 2). The Young’s modulus was in the range of 0.002 MPa to 0.009 MPa. The elongation at break was in the range of 81 to 273%, increasing with a decrease in the CHM content in the material.

## 4. Discussion

Increasing concern about the environmental impact of different polymer synthesis routes, especially those of polyurethanes, has prompted the search for new renewable and eco-friendly sources. The incorporation and use of bio-based monomers and the substitution of isocyanates is a promising route, and polyhydroxyurethanes (PHUs) have garnered attention, especially in biomedical applications. Therefore, this work aimed to synthesize non-isocyanate polyhydroxyurethanes from soybean oil, BDA, and CHM and characterize their physicochemical, mechanical, and thermal properties.

The results show that the modification of ESBO with carbon dioxide can generate CSBO which is necessary for the aminolysis reaction to obtain NIPHUs (Figure 1). Similar procedures have been performed by several authors in recent years. Patel et al. prepared CSBO at 750 psi (5.17 MPa) and 110 °C for 48 h and obtained the same results as this investigation. Nevertheless, the time used in this research was reduced by 24 h [28].

The reaction of the modified soybean oil with different combinations of BDA and CHM allowed us to obtain NIPHU films. Increasing the percentage of CHM in the matrix generated brittle NIPHUs, which broke during the demolding process; therefore, these NIPHUs were not considered for potential wound dressing applications. The results show that the inclusion of CHM improved the mechanical properties of these NIPHUs, but an excess did not lead to flexible materials.

In comparison to polyurethanes, PHUs present a higher water absorption limit due to the hydroxyl groups adjacent to the urethane linkages [29]. In this regard, the incorporation of CHM to the backbone of the NIPHUs increases their hydrophilicity (Figure 3b) but does not significantly affect water absorption. As reported in other investigations, the synthesis of NIPHUs by using BDA generates hydrophobic materials [28] Therefore, a moist environment is desired to create dressings that allow for better interaction with water. As expected, a direct correlation between the bulk hydrophilicity and the crosslink density of networks has been observed in previous research [6]. Due to the hydrophobic characteristics of the NIPHUs synthetized with carbonated soybean oil, the interaction between these materials and hydrolytic and oxidative media is low; in this study, there was no mass loss within the 21 days of the study, indicating the stability of the synthetized NIPHUs in relation to the time of wound healing. For would dressing applications, materials with a suitable water absorption rate are desired for their ability to absorb exudates and provide a moist environment suitable for the wound healing process, as well as a cooling sensation [30].

The thermal stability of the NIPHUs was evaluated with thermogravimetric analysis. All the NIPHUs presented thermal stability up to 200 °C, and weight loss was observed at temperatures under 100 °C, which might have been due to the moisture present in the samples. In agreement with other studies, the three stages of degradation could be attributed to the degradation of urethane linkages during the first stage, and then polymer degradation during the second and third stages [31]. The degradation of the soft segment of these NIPHUs is related to the CSBO structure.

Finally, in terms of mechanical properties, the tensile strength of the NIPHUs agrees with that of similar NIPHUs generated from CSBO. Patel et al. synthesized NIPHUs from CSBO and BDA with a tensile strength of 0.4878 MPa [28], compared to a tensile strength of 0.97 MPa of the NIPHU-100/0 in this study. Compared to polyurethanes synthesized for wound dressing applications, the tensile strength was in the range of 0.7 to 18 MPa for dermal cell culture [27], making them good candidates for wound dressings. Nevertheless, the mechanical properties of NIPHUs are lower when compared to conventional polyurethanes, such as those synthesized from vegetable oils like castor oil. Different blends of castor oil with aliphatic, cycloaliphatic, and aromatic isocyanates possess a maximum tensile strength of 16.86 MPa [32], while polyurethanes synthesized from carbonated soybean oil and isophorone diisocyanate have a tensile strength ranging from 5 to 17 MPa [6]. This remains a limitation in the application of NIPHUs to biological environments, but the inclusion of mixtures of different diamines could overcome this.

In this study, the inclusion of CHM in the formulation did not modify the tensile strength of the materials but influenced the elongation at break, increasing it from 81.13% to 273.21%. This increase influenced the flexibility of the dressing, which is an important key in the design for the comfort of patients [16]. Likewise, Gholami prepared a wound dressing from CSBO but with the inclusion of isocyanates, which reached an elongation at break between 140% and 390% [6]. These results show that the chemical or physical crosslinking in NIPHUs is not high enough to allow for the free movement of the chains, but the tensile strength is high enough to create a dressing for application on the wound zone, along with an appropriate water absorption rate to create an adequate moist environment for the progress of the wound healing stages.

## 5. Conclusions

A number of studies have been conducted, aiming to avoid the use of petrochemical monomers and to utilize eco-friendly synthesis routes for the synthesis of removable monomers. Soybean oil is a vegetable oil that can be modified with carbon dioxide to obtain novel wound dressings. The incorporation of 50% (molar percentage) of CHM in the NIPHU formulation (a cycloaliphatic diamine) increased the elongation at break from 81.13% to 273.21%, making the obtained NIPHUs more suitable for wound dressing applications without significant modifications to the physical, chemical, and thermal properties. NIPHUs obtained from vegetables oils, such as soybean oil, can be used in an eco-friendly manner for the fabrication of wound dressings that can replace existing PU dressings.

## Figures and Tables

**Table 1:** Composition of non-isocyanate polyhydroxyurethane films.

ID Sample	BDA (% Molar)	CHM (% Molar)
NIPHU-100/0	100	0
NIPHU-75/25	75	25
NIPHU-67/33	67	33
NIPHU-50/50	50	50
NIPHU-33/67	33	67
NIPHU-25/75	25	75
NIPHU-0/100	0	100

**Table 2:** Young’s modulus, tensile strength, and elongation at break of NIPHUs synthetized from CSBO with different contents of BDA and CHM. The data are expressed as the mean ± SD (*n* = 3).

Sample	Young’s Modulus (MPa)	Tensile Strength (MPa)	Elongation at Break (%)
NIPHU-100	0.009 ± 0.002 ^a^	0.97 ± 0.16 ^a^	81.13 ±18.82 ^a^
NIPHU-75/25	0.006 ± 0.001 ^a,b^	1.13 ± 0.05 ^a^	145.89 ± 39.69 ^a,b^
NIPHU-67/33	0.002 ± 0.001 ^b^	0.49 ± 0.22 ^a^	207.65 ± 31.05 ^b,c^
NIPHU-50/50	0.003 ± 0.0005 ^b,c^	0.96 ± 0.07 ^a^	273.21 ± 17.62 ^c^

According to the analysis of variance and Tukey’s pairwise comparison test, means that do not share the same letter are significantly different (*p* < 0.05).

## Data Availability

Data are contained within the article.

## References

[1] Wendels S., Avérous L. (2021). Biobased Polyurethanes for Biomedical Applications. Bioact. Mater..

[2] Gostev A.A., Karpenko A.A., Laktionov P.P. (2018). Polyurethanes in Cardiovascular Prosthetics. Polym. Bull..

[3] Mokhtari C., Malek F., Manseri A., Caillol S., Negrell C. (2019). Reactive Jojoba and Castor Oils-Based Cyclic Carbonates for Biobased Polyhydroxyurethanes. Eur. Polym. J..

[4] Gennen S., Grignard B., Thomassin J.M., Gilbert B., Vertruyen B., Jerome C., Detrembleur C. (2016). Polyhydroxyurethane Hydrogels: Synthesis and Characterizations. Eur. Polym. J..

[5] Carayon I., Szarlej P., Gnatowski P., Piłat E., Sienkiewicz M., Glinka M., Karczewski J., Kucińska-Lipka J. (2022). Polyurethane Based Hybrid Ciprofloxacin-Releasing Wound Dressings Designed for Skin Engineering Purpose. Adv. Med. Sci..

[6] Gholami H., Yeganeh H. (2020). Vegetable Oil-Based Polyurethanes as Antimicrobial Wound Dressings: In Vitro and in Vivo Evaluation. Biomed. Mater..

[7] Yang J.M., Huang Y.F., Dai J.J., Shi X.A., Zheng Y.Q. (2021). A Sandwich Structure Composite Wound Dressing with Firmly Anchored Silver Nanoparticles for Severe Burn Wound Healing in a Porcine Model. Regen. Biomater..

[8] Laurano R., Boffito M., Ciardelli G., Chiono V. (2022). Wound Dressing Products: A Translational Investigation from the Bench to the Market. Eng. Regen..

[9] Maaz M., Maqsood S., Gull N., Tabish T.A., Zia S., Ullah R., Muhammad S., Arif M. (2021). Polymer-Based Biomaterials for Chronic Wound Management: Promises and Challenges. Int. J. Pharm..

[10] Zhang C., Yang X., Yu L., Chen X., Zhang J., Zhang S., Wu S. (2024). Electrospun Polyasparthydrazide Nanofibrous Hydrogel Loading with In-Situ Synthesized Silver Nanoparticles for Full-Thickness Skin Wound Healing Application. Mater. Des..

[11] Samatya Yılmaz S., Aytac A. (2023). The Highly Absorbent Polyurethane/Polylactic Acid Blend Electrospun Tissue Scaffold for Dermal Wound Dressing. Polym. Bull..

[12] Haniffa M.A.C.M., Munawar K., Ching Y.C., Illias H.A., Chuah C.H. (2021). Bio-Based Poly(Hydroxy Urethane)s: Synthesis and Pre/Post-Functionalization. Chem. Asian J..

[13] Carré C., Ecochard Y., Caillol S., Avérous L. (2019). From the Synthesis of Biobased Cyclic Carbonate to Polyhydroxyurethanes: A Promising Route towards Renewable Non-Isocyanate Polyurethanes. ChemSusChem.

[14] Mubofu E.B. (2016). Castor Oil as a Potential Renewable Resource for the Production of Functional Materials. Sustain. Chem. Process..

[15] Yang X., Wang S., Liu X., Huang Z., Huang X., Xu X., Liu H., Wang D., Shang S. (2021). Preparation of Non-Isocyanate Polyurethanes from Epoxy Soybean Oil: Dual Dynamic Networks to Realize Self-Healing and Reprocessing under Mild Conditions. Green Chem..

[16] Gholami H., Yeganeh H. (2021). Soybean Oil-Derived Non-Isocyanate Polyurethanes Containing Azetidinium Groups as Antibacterial Wound Dressing Membranes. Eur. Polym. J..

[17] Guzmán A.F., Echeverri D.A., Rios L.A. (2017). Carbonation of Epoxidized Castor Oil: A New Bio-Based Building Block for the Chemical Industry. J. Chem. Technol. Biotechnol..

[18] Raden Siti Amirah H., Ahmad Faiza M., Samsuri A. (2013). Synthesis and Characterization of Non-Isocyanate Polyurethane from Epoxidized Linoleic Acid—A Preliminary Study. Adv. Mat. Res..

[19] Uscátegui Y.L., Díaz L.E., Valero M.F. (2019). In Vitro and in Vivo Biocompatibility of Polyurethanes Synthesized with Castor Oil Polyols for Biomedical Devices. J. Mater. Res..

[20] Mi H.Y., Jing X., Hagerty B.S., Chen G., Huang A., Turng L.S. (2017). Post-Crosslinkable Biodegradable Thermoplastic Polyurethanes: Synthesis, and Thermal, Mechanical, and Degradation Properties. Mater. Des..

[21] Weems A.C., Wacker K.T., Carrow J.K., Boyle A.J., Maitland D.J. (2017). Shape Memory Polyurethanes with Oxidation-Induced Degradation: In Vivo and in Vitro Correlations for Endovascular Material Applications. Acta Biomater..

[22] (2015). Standard Test Method for Tensile Properties of Plastics.

[23] Ahmad Z.R., Mahanwar P.A. (2022). Synthesis and Properties of Foams from a Blend of Vegetable Oil Based Polyhydroxyurethane and Epoxy Resin. Cell. Polym..

[24] Wang T., Deng H., Li N., Xie F., Shi H., Wu M., Zhang C. (2022). Mechanically Strong Non-Isocyanate Polyurethane Thermosets from Cyclic Carbonate Linseed Oil. Green Chem..

[25] Ahmad Zubir S., Mat Saad N., Harun F.W., Ali E.S., Ahmad S. (2018). Incorporation of Palm Oil Polyol in Shape Memory Polyurethane: Implication for Development of Cardiovascular Stent. Polym. Adv. Technol..

[26] Buffa J.M., Mondragon G., Corcuera M.A., Eceiza A., Mucci V., Aranguren M.I. (2018). Physical and Mechanical Properties of a Vegetable Oil Based Nanocomposite. Eur. Polym. J..

[27] Mistry P., Chhabra R., Muke S., Narvekar A., Sathaye S., Jain R., Dandekar P. (2021). Fabrication and Characterization of Starch-TPU Based Nanofibers for Wound Healing Applications. Mater. Sci. Eng. C.

[28] Patel P., de Souza F.M., Gupta R.K. (2024). Study of Soybean Oil-Based Non-Isocyanate Polyurethane Films via a Solvent and Catalyst-Free Approach. ACS Omega.

[29] Hu S., Chen X., Torkelson J.M. (2022). Isocyanate-Free, Thermoplastic Polyhydroxyurethane Elastomers Designed for Cold Temperatures: Influence of PDMS Soft-Segment Chain Length and Hard-Segment Content. Polymer.

[30] Almasian A., Najafi F., Eftekhari M., Ardekani M.R.S., Sharifzadeh M., Khanavi M. (2020). Polyurethane/Carboxymethylcellulose Nanofibers Containing Malva Sylvestris Extract for Healing Diabetic Wounds: Preparation, Characterization, in Vitro and in Vivo Studies. Mater. Sci. Eng. C.

[31] Cornille A., Serres J., Michaud G., Simon F., Fouquay S., Boutevin B., Caillol S. (2016). Syntheses of Epoxyurethane Polymers from Isocyanate Free Oligo-Polyhydroxyurethane. Eur. Polym. J..

[32] Uscategui Y.L., Diaz L.E., Gomez-Tejedor J.A., Valles-Lluch A., Vilarino-Feltrer G., Serrano M.A., Valero M.F., Uscátegui Y.L., Díaz L.E., Gómez-Tejedor J.A. (2019). Candidate Polyurethanes Based on Castor Oil (Ricinus Communis), with Polycaprolactone Diol and Chitosan Additions, for Use in Biomedical Applications. Molecules.

